# Editorial: Surgical approaches for the treatment of glioma

**DOI:** 10.3389/fonc.2022.1060539

**Published:** 2022-11-03

**Authors:** G. La Rocca, E. Mazzucchi, G. Sabatino, GM. Della Pepa, A. Olivi

**Affiliations:** ^1^ Institute of Neurosurgery, Fondazione Policlinico Universitario A. Gemelli IRCCS, Catholic University, Rome, Italy; ^2^ Unit of Neurosurgery, Mater Olbia Hospital, Olbia, Italy

**Keywords:** glioma, brain surgery, glioblastoma, high grade glioma (HGG), low grade glioma (LGG)

Advances in the field of technology and molecular biology have improved the treatment of glioma. The concept of maximal safe resection is the main objective of surgery for this complex disease. To achieve this, researchers have developed several strategies. In addition to technical and manual ability, four points emerge as fundamental to provide the best care in surgical treatment of gliomas: technology, decision making, anatomy, laboratory research.

Technology may be an ally for the surgeon in recognizing the tumour and surrounding eloquent areas. Intraoperative imaging can, as a matter of fact, allows real-time identification of tumour residual and prevent complication. Hou and Tang and Hou et al. proposed a wise integration of intraoperative ultrasound and intraoperative MRI, in accordance with a flourishing literature regarding the utility of integration of multiple techniques (1–3).

Indication and timing for surgical treatment, intraoperative strategy, when to stop resection: decision making is sometimes very difficult. In case of repeated surgery for glioma the indication for timing of reoperation and the use awake monitoring in these patients is discussed in an interesting review by Duffau.

Knowledge of anatomical restraints of resection and identification of areas at risk may be helped by technology and, when indicated, by awake or asleep mapping (4), but profound study of surgical anatomy even in anatomy laboratory (5), is nevertheless fundamental for the surgeon. Gong et al. present their experience with cingulate gyrus glioma and propose a classification, that could be useful in clinical practice.

Laboratory research is nevertheless fundamental in these tumours, in which surgical treatment is often insufficient. New perspectives for the intraoperative identification and treatment of glioma come from the work of Mander et al. who tested the efficacy of a tumour-targeting cell-penetrating peptide in an animal model of glioblastoma. Treatment of gliomas is increasingly based on the genetic and epigenetic mutations of the tumour. Intramedullary astrocytomas have specific frequency and types of mutations, often different from cranial gliomas. Hersh et al. performed a review of the relative literature.

Anatomy and pre-clinical research represent the fundament for the advances of surgical treatment of gliomas; technical and manual ability, aided by a wise use of technological tool and the ability to make the right decision, is the way to adequately treat each single patient in the most appropriate way.

## Author contributions

GL and EM drafted the article, revised it critically for important intellectual content. GD, GS and AO agreed to be accountable for all aspects of the work in ensuring that questions related to the accuracy or integrity of any part of the work are appropriately investigated and resolved. All authors contributed to the article and approved the submitted version.

## Conflict of interest

The authors declare that the research was conducted in the absence of any commercial or financial relationships that could be construed as a potential conflict of interest.

## Publisher’s note

All claims expressed in this article are solely those of the authors and do not necessarily represent those of their affiliated organizations, or those of the publisher, the editors and the reviewers. Any product that may be evaluated in this article, or claim that may be made by its manufacturer, is not guaranteed or endorsed by the publisher.

## References

[1] Mazzucchi E La Rocca G Ius T Sabatino G Della Pepa GM. Multimodality imaging techniques to assist surgery in low-grade gliomas. world neurosurgery. Gennaio (2020) 133:423–5. doi: 10.1016/j.wneu.2019.10.120 31881559

[2] Mazzucchi E La Rocca G Hiepe P Pignotti F Galieri G Policicchio D. Intraoperative integration of multimodal imaging to improve neuronavigation: a technical note. World Neurosurg (2022). 164:330–40. S1878-8750(22)00773-2. doi: 10.1016/j.wneu.2022.05.133 35667553

[3] Della Pepa GM Ius T La Rocca G Gaudino S Isola M Pignotti F. 5-aminolevulinic acid and contrast-enhanced ultrasound: The combination of the two techniques to optimize the extent of resection in glioblastoma surgery. Neurosurgery (2020). 86:E529-40. doi: 10.1093/neuros/nyaa037 32186345

[4] Ius T Mazzucchi E Tomasino B Pauletto G Sabatino G Della Pepa GM. Multimodal integrated approaches in low grade glioma surgery. Sci Rep (2021) 11(1):9964. doi: 10.1038/s41598-021-87924-2 33976246 PMC8113473

[5] La Rocca G Mazzucchi E Pignotti F Galieri G Rinaldi P Sabatino G. Advanced dissection Lab for neuroanatomy training. Front Neuroanat (2021) 15:778122. doi: 10.3389/fnana.2021.778122 35069130 PMC8769374

